# Ligand Similarity Complements Sequence, Physical Interaction, and Co-Expression for Gene Function Prediction

**DOI:** 10.1371/journal.pone.0160098

**Published:** 2016-07-28

**Authors:** Matthew J. O’Meara, Sara Ballouz, Brian K. Shoichet, Jesse Gillis

**Affiliations:** 1 Department of Pharmaceutical Chemistry, University of California San Francisco, San Francisco, California, 94158–2550, United States of America; 2 Cold Spring Harbor Laboratory, Stanley Institute for Cognitive Genomics, 500 Sunnyside Boulevard, Woodbury, NY, 11797, United States of America; Indiana University, UNITED STATES

## Abstract

The expansion of protein-ligand annotation databases has enabled large-scale networking of proteins by ligand similarity. These ligand-based protein networks, which implicitly predict the ability of neighboring proteins to bind related ligands, may complement biologically-oriented gene networks, which are used to predict functional or disease relevance. To quantify the degree to which such ligand-based protein associations might complement functional genomic associations, including sequence similarity, physical protein-protein interactions, co-expression, and disease gene annotations, we calculated a network based on the Similarity Ensemble Approach (SEA: sea.docking.org), where protein neighbors reflect the similarity of their ligands. We also measured the similarity with functional genomic networks over a common set of 1,131 genes, and found that the networks had only small overlaps, which were significant only due to the large scale of the data. Consistent with the view that the networks contain different information, combining them substantially improved Molecular Function prediction within GO (from AUROC~0.63–0.75 for the individual data modalities to AUROC~0.8 in the aggregate). We investigated the boost in guilt-by-association gene function prediction when the networks are combined and describe underlying properties that can be further exploited.

## Introduction

To interpret biological function, proteins are often organized into networks derived from large scale experimental observations including those measuring sequence similarity [1], physical interaction [2, 3], or mRNA co-expression [4, 5]. From these networks, inferences are made regarding specific molecular activity and their evolutionary origin, etiology of phenotype and disease, and response to therapeutic intervention. These inferences suggest specific hypotheses that can be tested in the lab and clinic.

Recently, large-scale protein-ligand databases, derived mostly from the medicinal chemistry literature, have become available [6, 7]. An example is the ChEMBL database, which now contains over 13 million activities linking over ten thousand targets with over 1.7 million compounds. The scale of these associations enable the calculation of protein networks that capture the chemical similarity of the ligand-addressed proteome; in such networks, two proteins are linked if they share similar ligands, where similarity may be measured by several widely-used chemoinformatics metrics [8–10]. There is an inference that if their sets of ligands are similar, that is, if the proteins are neighbors in the ligand-articulated networks, then it should be possible to find specific molecules that will bind to both members of the pair, even if none are known to do so. These networks have been successfully used to predict drug side-effect targets on a large scale [11–13], to seed drug repurposing [14], and to predict drug and reagent mechanism of action [12, 14–17], indicating ligand similarity reflects fundamental protein ligand binding preferences.

Here we investigate how, and whether, the inferences from functional genomic networks and from ligand similarity networks might complement functional genomic networks. Specifically we consider (1) the extent to which the networks contain distinct information and (2) whether combining the two sorts of networks adds inferential power. To do so, we build proteome scale networks for three functional genomic methods, and a ligand similarity network, and measured their correlation over their intersection of 1,131 human proteins. We then investigate whether adding the ligand similarity network improves gene function prediction using a guilt-by-association predictor of Gene Ontology annotations.

## Results

### Calculating ligand-based similarities

We compared proteins by the similarity of their ligands, as annotated in the ChEMBL database [6]. To overcome the fact that most ligands are only tested at a single target (65%), we use standard measures of ligand-ligand similarity based on shared patterns of atom and bonds to infer similarities between proteins that share no ligands (said another way, this enables targets that share no single ligand in common to still be found similar because their ligands are nevertheless related). The ligand similarity between two proteins is based on the sum of pairwise similarities among the sets of their ligands—for instance, for two proteins each with 100 ligands, 10,000 pairwise similarities are calculated and summed. This sum is compared to the sum one would expect for random sets of ligands, drawn from ChEMBL, using the BLAST statistical machinery; this is the essence of the Similarity Ensemble Approach (SEA) [11]. As in the more familiar sequence comparisons, ligand-based similarities were expressed as expectation values (E-values), representing the likelihood that the pair of proteins would have the ligand set similarity that they do, compared to random.

We selected 1,131 metazoan targets from ChEMBL16 [6] that had at least 5 annotated ligands (median of 41, max 3,688), could be mapped to human targets using HomoloGene [18], and were present in both co-expression (CoExp) and protein-protein interaction (PPI) networks of human genes. Among these were 109 GPCRs, 58 ion channels, 27 NHRs, 29 transporters, 353 kinases, and 393 other enzymes; all major classes of drug targets were represented in this set. Of the 639,015 pairs of targets compared, 47,329 were calculated to have ligand-based (SEA) E-values of 1e-5 or better, with 18,284 having E-values 1e-20 or better. The 591,686 pairs that had E-values worse than 1e-5 were considered unrelated by ligand similarity. Of the possible 62,128 associations among the kinases, 34,737 (55.9%) are significant, representing a total of 73% the significant associations in the LigNet network. This bias is present in the whole SEA network as 435 of the 2,920 targets are kinases (14.8%) and 650,638 associations among kinases 155,135 (27.6%) are significant, where only 2.2% would be expected at random. It reflects both the deep pharmacological exploration of the kinase family [19] relative to the recent expansion to genome scale pharmacology [20, 21], and the high conservation of the ATP-binding site leading to the recognition of chemically similar ligands among kinases.

### Comparing ligand similarities to sequence similarities

**Fig 1:**
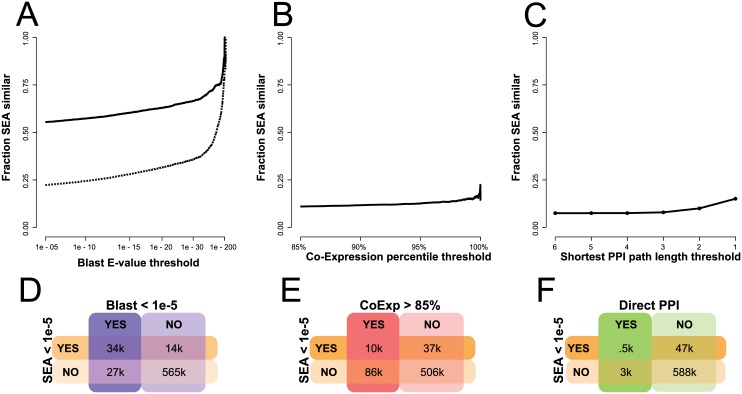
Discordance of bioinformatics and functional genomic similarity with chemoinformatic similarity. Likelihood that two proteins will be related by ligand similarity (solid line: SEA E-value < 1e-5, dashed line: SEA E-value < 1e-20) given a threshold in the (A) sequence similarity network, (B) co-expression network, and (C) extended protein-protein interaction network. The Y-axis is the likelihood that pairs of targets will have a SEA E-value better than 1e-5 (and, for sequence similarity, also 1e-20) at any given threshold of similarity on the X-axis. (D-F) Truth tables showing the correspondence of the protein-protein pairs that either are or are not related by ligand similarity *and* by sequence similarity, co-expression, or direct protein-protein interactions. In the upper left and lower right squares, the ligand-based and genomics association agree that the targets are or are not related, while in the lower left and upper right they disagree.

We measured the sequence similarity of these protein pairs, and compared these to those measured by SEA for ligands. Of the about 60,819 pairs of targets that have BlastP E-values of 1e-5 or better, 33,705 are also related by ligand similarities of 1e-5 or better, but a similar number, 27,114, are unrelated by ligand similarity (Fig 1A). For example, both the nociceptin opioid receptor vs. the other Mu, Kappa, and Delta opioid receptors, and vascular endothelial vs. platelet-derived growth factor receptors [22] pairs are highly sequence similar (BlastP E-value < 1e-86) and highly ligand similar (SEA E-value < 1e-50), while both the N- and L-type voltage gated calcium channels vs. the bile acid and oxysterol nuclear hormone receptors [23] pairs are highly sequence similar but recognize unrelated ligands.

Correspondingly, there are 13,624 other pairs of proteins that had related ligand sets but were unrelated by sequence. Since 1e-5 is an arbitrary, though arguably generous BlastP E-value cut-off, we calculated the likelihood that a pair of proteins that were related by sequence would also be related by ligand similarity over the full range of sequence-based E-values (Fig 1A). For most sequence-related proteins, the likelihood that they also bind similar ligands is only about 55%. Though this rises with sequence identity, it does so slowly, and the likelihood of sharing similar ligand sets only reaches 80% by BlastP E-values of 1e-86. If one were to use a more stringent—but by no means extreme—ligand-based E-value of 1e-20, then the 80% confidence point that sequence-related proteins will share related ligand sets is only reached at BlastP E-values of 1e-180.

If such baroque E-values illustrate the limited ability of sequence similarity to confidently predict ligand similarity between targets, there are nevertheless proteins for which ligands may be predicted based on sequence similarity. At the 1e-180 E-value cutoff, there are 348 human proteins for which there are no annotated ligands in ChEMBL that one is 80% confident will share ligand similarity with a target for which ligands are known (S1 Table, S1 Fig). For example, the betaine transporter (SLC6A12) has only 3 potent ligands in ChEMBL but is highly sequence similar to the GABA transporter (SLC6A1), which has 70 annotated ligands. Similarly, the epigenetic demethylase KDM4D has no ligands annotated to it, but the sequence similar KDM4E has several thousand. KDM4E has ligands at least partly because it has an assay adapted to high-throughput screening. However, it is KDM4D that arguably has been associated with the more interesting biology [24]. For pairs of targets like this, the extension of ligands from one target, if far from infallible, is straightforward, with candidate ligands often readily available to test [6, 25]. This analysis suggests sequence similarity is moderate predictor of ligand similarity with a substantial number of sequence dissimilar proteins having strong ligand similarity.

### Comparing ligand similarities to functional genomic similarities

We undertook the same comparisons of ligand-based similarities to similarities in co-expression and protein-protein interactions (Fig 1 panels **B** and **C**). We constructed a weighted co-expression network (CoExp) by aggregating data from 42 individual experiments totaling 940 samples (see Methods). As with sequence similarity, whereas there is a significant trend for proteins that are among the top 0.5% of co-expressing pairs to share related ligands, (χ^2^ = 1.3e3, p < 2.2e-16), pragmatically the effect size is marginal and only 9.3% of the top 15% of co-expressed pairs show any SEA interactions at all (Fig 1B). Within the PPI network derived from BioGRID [26], 3,850 pairs were reported to physically interact. Of these, 581 were related by ligand similarity with E-values of 1e-5 or better. To model indirect connections [27], we extend the network by adding edges between node pairs with minimum path length less than seven, weighted by the inverse of the minimum path length. As with co-expression, there is a high discordance between this extended PPI network (ExtPPI) and the ligand-based SEA network (LigNet), with the tendency for PPI interactors to be SEA interactors being statistically significant but with little overlap, with only 3.6% of the ExtPPI interactors sharing similar ligand sets (Fig 1C). The level of discordance remains even when compared against co-expression and PPI networks constructed with different curation and inclusion criteria (S2 Fig, average AUROC = 0.54).

### Comparing ligand and functional genomic associations to the Gene Ontology

This discordance is also reflected by the GO term annotations predicted by the functional genomics- and ligand-based associations. The per-term enrichments for each network are comparable because the analysis is performed over a common set of proteins and the GO terms annotating them (Table 1). PPI and co-expression associations usually reflect biological processes in GO, such as DNA replication, transcription, and mitosis, which involve coordinated actions of multiple proteins, often in complexes (Table 1, highlighted in green). Conversely, ligand-based associations between targets often enrich for functions associated with particular proteins, such as kinases, peptidases, and GPCRs, reflecting molecular functions in GO such as metabolism and signaling (Table 1, highlighted in pink).

**Table 1:** Top 20 performing GO terms in the different networks.

Co-expression		Extended PPI		SEA Network	
GO Term	AUROC	GO Term	AUROC	GO Term	AUROC
nucleobase metabolic process	0.86	DNA-dependent transcription, initiation	0.95	protein kinase activity	0.95
DNA replication	0.82	transcription initiation from RNA polymerase II promoter	0.94	metallopeptidase activity	0.95
leukocyte activation	0.80	transcription from RNA polymerase II promoter	0.92	kinase activity	0.95
cell cycle phase	0.80	transcription regulatory region DNA binding	0.92	transferase activity, transferring phosphorus-containing groups	0.95
ion gated channel activity	0.79	transcription, DNA-dependent	0.91	phosphotransferase activity, alcohol group as acceptor	0.95
M phase	0.79	Toll signaling pathway	0.91	peptidase activity, acting on L-amino acid peptides	0.94
substrate-specific channel activity	0.79	TRIF-dependent toll-like receptor signaling pathway	0.91	endopeptidase activity	0.93
mitotic cell cycle phase transition	0.79	toll-like receptor 4 signaling pathway	0.91	protein serine/threonine kinase activity	0.92
regulation of purine nucleotide biosynthetic process	0.79	regulatory region nucleic acid binding	0.91	serine-type peptidase activity	0.91
synaptic transmission	0.79	toll-like receptor signaling pathway	0.91	nucleobase metabolic process	0.91
gated channel activity	0.79	toll-like receptor 3 signaling pathway	0.91	protein tyrosine kinase activity	0.91
ion channel activity	0.79	pattern recognition receptor signaling pathway	0.91	peptidase activity	0.90
positive regulation of I-kappaB kinase/NF-kappaB cascade	0.79	innate immune response-activating signal transduction	0.91	transmembrane receptor protein tyrosine kinase activity	0.90
cell chemotaxis	0.78	nucleic acid binding transcription factor activity	0.91	serine-type endopeptidase activity	0.90
chromosome organization	0.78	regulatory region DNA binding	0.90	phosphorylation	0.90
interphase	0.78	sequence-specific DNA binding transcription factor activity	0.90	serine hydrolase activity	0.90
multicellular organismal signaling	0.78	JNK cascade	0.90	protein phosphorylation	0.90
G-protein coupled receptor signaling pathway, coupled to cyclic nucleotide second messenger	0.78	MyD88-dependent toll-like receptor signaling pathway	0.90	protein autophosphorylation	0.89
neurological system process	0.78	toll-like receptor 2 signaling pathway	0.89	transmembrane receptor protein kinase activity	0.88
transmission of nerve impulse	0.78	toll-like receptor 1 signaling pathway	0.89	stress-activated MAPK cascade	0.87

Biological process (green) and molecular function (pink).

**Fig 2:**
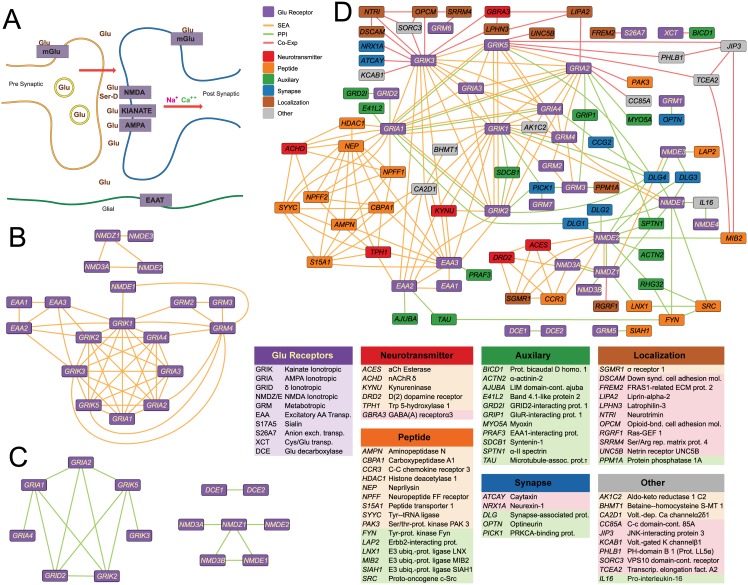
Functional genomic and chemoinformatic interactions among proteins involved in glutamate signaling. (**A**) A schematic of glutamate signaling showing three of the major types of proteins involved—ionotropic glutamate receptors, metabotropic glutamate receptors, and glutamate transporters. (**B**) Proteins annotated in IUPHAR as involved in glutamatergic neurotransmission and linked by ligand-based SEA E-values better than 1e-25. (**C**) Proteins annotated in IUPHAR as involved in glutamatergic neurotransmission and linked by human physical protein-protein interactions from BioGRID (v3.2.121). (**D**) The ligand-similarity and PPI networks from (**B**) and (**C**) merged and extended to adjacent related proteins, using (1) ligand-based (orange edges, using a SEA E-value threshold of 1e-75), (2) protein-protein interactions restricting to those supported by at least one low-throughput observation, one physical observation, and observed in at least two different experiments (green edges), and (3) co-expression links at a 94% threshold (pink edges). Edges that overlapped between shared nodes from the independent networks shown in (**B**) and (**C**), calculated at less stringent cut-offs, are preserved here to illustrate the few cases of overlap between the networks (e.g., between GRIA1 and GRIA2).

These differences between the chemical and proteomic views are also apparent in particular functional networks. For instance, consider the network seeded with the 45 non-mitochondrial glutamate signaling proteins annotated in IUPHAR (www.guidetopharmacology.org) (Fig 2A). At a relatively stringent SEA E-value cutoff of 1e-25, 19 of these 45 receptors are connected, with links spanning the glutamate ionotropic and metabotropic receptors, and the glutamate transporters (Fig 2B). In contrast, the PPI network is mostly restricted to edges within the ionotropic receptors (Fig 2C) of which 14 have at least one connection, typically with much smaller degree than that in the ligand-based network. None of the 45 glutamate receptors are significantly co-expressed, and thus do not form a module on their own.

The independence of the three networks is made clearer by extending them to neighboring proteins, outside of formal glutaminergic signaling but connected to at least one of the 45 glutaminergic proteins (Fig 2D). Thresholds were chosen to get comparable number of interactions; SEA E-value < 1e-75, co-expression > 94%, and PPI direct connections from BioGRID (v3.2.121) observed in a low throughput, physical experiment and by at least two different methods. While the ligand similarity and PPI criteria are stringent, the co-expression threshold here is permissive, as 99% is commonly used [5].

The networks show striking orthogonality, with few inter-network edge overlaps and clear intra-network clustering (Fig 2D). While this can be partially attributed to the stringent thresholds and to experimental limitations (e.g. the ionotropic receptors form hetero-tetramers that should be captured in the PPI and co-expression networks, but are missed), it mostly reflects the different peripheral receptors captured by the different associations. For example, ligand similarity connects other neurotransmitter signaling proteins including a dopamine receptor (DRD2), a serotonin biosynthesis enzyme (TPH1), an acetylcholine receptor and acetylcholinesterase (ACHD and ACES), and kynureninase (KYNU). Conversely, PPI network extends into auxiliary modulators of glutamate transporters (e.g., PRAF1 with EAA3) and glutamate receptors (PICK1, DLG1-4, GRIP1, and CCG2). The co-expression networks connect axon guidance proteins (DSCAM, UNC5B, and NTRI) and synapse and adhesion proteins (LPHN3, NTRI, FREM2, LIPA2, OPCM) related to the internal network apparatus rather than, as in the ligand-based network, the proteins acting on or responding to the chemical information that is transmitted. The SEA network is both topologically orthogonal from the co-expression and PPI networks, but also capture functionally dissimilar associations.

### Characterizing gene function from ligand similarities

**Fig 3:**
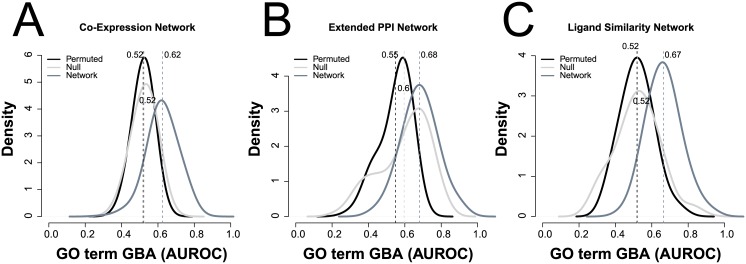
Ligand-based networks better recapitulate Gene Ontology than do PPI or co-expression networks. The (**A**) co-expression network and the (**B**) extended protein-protein interaction network are compared with the (**C**) ligand derived network for their ability to characterize gene function (defined in the Gene Ontology, GO). We assessed performance through cross-validation (area under the ROC curve, AUROC) of a neighbor-voting algorithm. Each curve represents the distribution of AUROCs across 790 GO terms. The dark grey shows the scores in cross-validation in each network, the black curves are the AUROCs after permuting the network nodes, while the light gray curves are the scores using the node degree as a generic predictor across all functional categories. The PPI network has the highest performance (**B**, dark grey, AUROC = 0.68) but this reflects node degree bias (light grey line, AUROC = 0.6). Co-expression has less bias (**A**, light grey line, AUROC = 0.52), but performs less well (dark grey line, AUROC = 0.62). The ligand network performs almost as well (**C**, dark grey line, AUROC = 0.67) as the extended PPI network with little node degree bias (light grey line, AUROC = 0.52). The random permutation of each network (black), have AUROCs between 0.48 and 0.5.

To systematically assess the complementarity between ligand-based and proteomic networks for gene function prediction, we adapted a machine learning approach, neighbor-voting guilt-by-association (Methods), in which genes are predicted to participate in a given function based on the fraction of their neighbors that participate in that function. Briefly, for each term within the Gene Ontology (GO), we predicted held-back (validation) gene membership from known (training) genes within it. We measured this cross-validation performance for each GO term by the area under the receiver operating characteristic (AUROC) curve, which plots the false positive rate vs. true positive rate. The AUROC gives the probability that a positive (correct gene-function) is ranked above a negative (incorrect gene-function); thus, 0.5 is random, 0.7 is generally considered good, and 0.9 is exceptional for any given problem. Because we are predicting all GO terms using a comparatively simple learner, this represents a conservative lower bound on performance. All three networks are significantly predictive with the ligand similarity network being nearly as predictive as the ExtPPI network and more predictive than the CoExp network by ROC, with AUROCs of LigNet = 0.67, ExtPPI = 0.68, and CoExp = 0.62 (Fig 3). However, there is divergence in the functional properties well characterized by genomics- vs. ligand-based associations (Table 1, S2 Table). PPI and co-expression associations usually reflect biological processes in GO, such as DNA replication, transcription, and mitosis, which involve coordinated actions of multiple proteins, often in complexes (Table 1, highlighted in green). Alternatively, ligand-based associations between targets often enrich for functions associated with particular proteins, such as kinases, peptidases, and GPCRs, reflecting molecular functions in GO such as metabolism and signaling (Table 1, highlighted in pink).

One potential confound in predicting functions for genes is that some hub genes, such as tumor suppressor p53, ubiquitin, or proto-oncogene c-Src, are associated with many functions, and correctly predicting these multifunctional genes can dominate performance [28]. Such bias can be misleading because it can obscure the inability to predict the function of non-hub genes. To characterize the fraction of performance simply due to this fact, for each network we ranked genes by their node degree (number of interactions) and used it as a generic predictor across all functional categories. Consistent with previous observations, predicting a gene’s function by its node degree in the ExtPPI network has an AUROC of 0.6, indicating a strong degree bias, which the CoExp and LigNet networks do not share (AUROC values of 0.53 and 0.52). Thus, the LigNet network is both relatively high performing and comprehensive, without being dominated by any particular hub gene (this is explored in more detail below and in S3 Fig).

**Fig 4:**
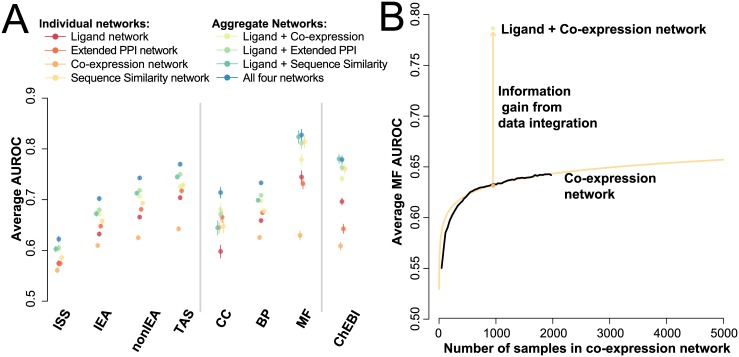
Improving performance of the network as measured through Guilt-by-Association on GO. (**A**) The prediction of GO annotation terms grouped by evidence code and sub-ontology by individual and combined networks. The ChEBI subset consists of terms associated with the Chemical Entities of Biological Interest (ChEBI) ontology (S3 Table, S3 Fig). Error bars represent the standard error of the mean. Combining the networks improves performance substantially (average ~0.80). (**B**) The CoExp network performance gains with increasing sample size but with diminishing returns, especially when compared with the gains obtained by combining the orthogonal chemoinformatics network. Extrapolating the aggregation curve (orange line), we predict that we would need millions of more samples to achieve similar performance with CoExp alone as with the combined chemoinformatics and CoExp networks (orange arrow).

Given the variable quality of GO annotations, more weight should be given to the correct prediction of annotations with strong supporting evidence. Accordingly, we partitioned annotations by the standard GO evidence codes: Traceable Author Statements (TAS, most reliable), Inferred from Sequence Similarity (ISS, least reliable), Electronic Annotation (IEA), and all others (nonIEA), and repeated the analysis (Fig 4A). The LigNet network performance improves substantially over TAS annotations (p < 3.74e-13, AUROC~0.70), with even greater performance when we focus on the molecular function GO terms (AUROC~0.75) and terms associated with the ChEBI ontology (AUROC~0.70). The ExtPPI network rises more modestly from its higher baseline in the TAS annotations (AUROC~0.72). However, over ISS annotations, the LigNet network loses almost 100% of its performance, falling to values little better than random (AUROC~0.57), in contrast to a less extreme decline in the ExtPPI (AUROC~0.57) and co-expression data (AUROC~0.56). In summary, where we believe functional annotation to be strongest, inferring function from ligands works best and where function is derived from sequence, it works worst, again speaking to the complementarity of the sequence, functional genomics, and ligand-similarity views.

The ligand-based network thus seems topologically distinct from other networks and by itself performs well in predicting known functional annotations, and so we investigated whether the ligand network could improve function prediction in conjunction with other networks. We constructed two aggregate networks, the CoExp network and the ExtPPI network each combined with the LigNet network (see Methods) and see guilt-by-association performance of AUROC = 0.71 and AUROC = 0.72 respectively, improving on the performance of individual networks. In MF derived functions, this rises to AUROC = 0.78 and AUROC = 0.81 (S3 Table). This improvement is particularly striking as the performance of the CoExp network has diminishing returns as sample size grows [29] (Fig 4B), suggesting that no reasonable amount of new expression data could compensate for the medicinal chemistry data captured by SEA. This analysis suggests the LigNet network is not only orthogonal to the CoExp and ExtPPI networks, but also brings useful information.

### Robustness and functional localization in gene function prediction

Given the positive results over the selected subset of genes and GO term inference, a critical question is the extent to which the performance is robust with respect to the evaluation metric and the localization of function to particular genes within the networks. These factors determine the efficacy of making specific testable predictions and the ability to generalize to new contexts.

Appropriate metrics for evaluation in gene function prediction [30] and machine learning more generally [31] remain ongoing areas of active research as different metrics capture different aspects of the performance. Area under the ROC curve can be interpreted as a rescaled Mann-Whitney test statistic, where the null is that the medians of positives and negatives are the same [32]. This interpretation highlights that AUROC rewards rank improvements in low and high ranking predictions equally. This uniform weighting of changes in gene ranks within the AUROC makes it a useful evaluation metric since changes in performance are likely to “sum” in an intuitive way across predictors or tasks. However, the AUROC may be less useful if we do not care if all genes have modestly improved in performance but care quite a lot if one of the very top predictions is a positive, as in many cost-limited experiments. For this latter consideration, average precision is useful since it weights positives near the top more heavily (see formula in methods). It also conveniently maps onto experimental requirements since 1/(average precision) represents the number of genes that would need to be tested before a “hit” would be obtained, on average [33]. However, weighting top predictions highly runs the risk of being strongly influenced by outliers, which may reflect overfitting through data contamination (e.g., GO annotations originally derived from BLAST in IEA terms being “predicted” by BLAST). Since precision-recall will reveal both useful and potentially deleterious outliers more readily, we re-evaluated our networks using it as a performance metric

**Fig 5:**
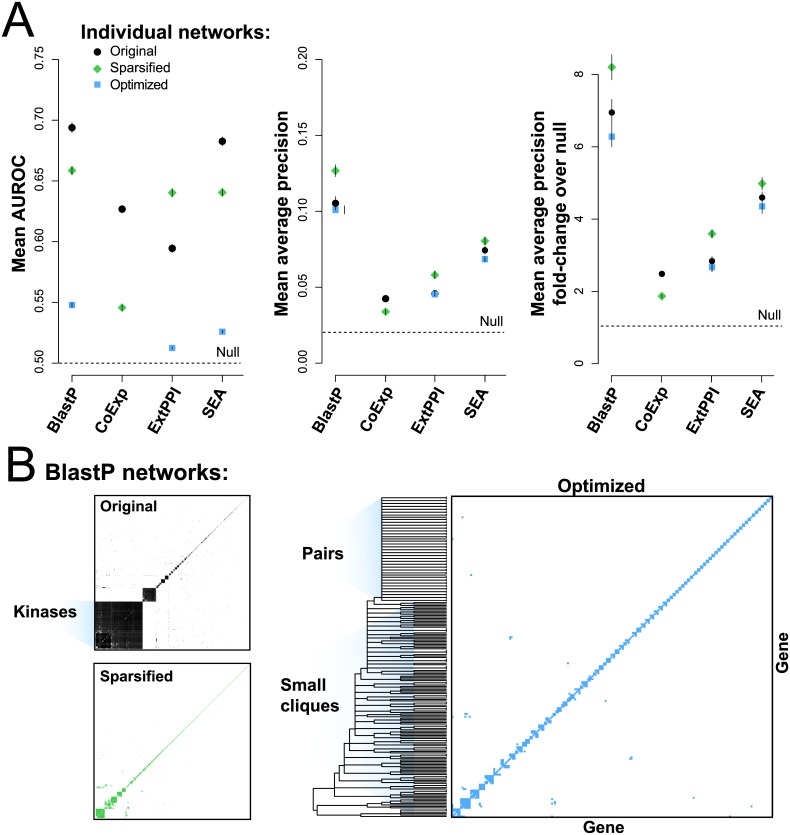
Experimental characterization to test robustness in methods and underlying data. (**A**) Performance of the networks as measured by the AUROC, average precision and average fold change precision. The black points represent the full network as assessed on a GO subset. The green points show the performance of the 1% sparsified network, while the blue the optimized version. (**B**) The topology of the BlastP sequence similarity network. The original, once optimized, has few connections, mostly pairs and cliques.

To begin, we re-evaluated our networks for prediction of the propagated non-IEA GO term annotations reporting the mean average precision (MAP). The null performance is the mean of the fraction of positive genes for each term (in the held-out data) and equal to 0.020. We find the MAP scores broadly support our original observations with the ligand network achieving 0.074, and the remaining networks showing consistent relative performances (BlastP = 0.10, CoExp = 0.042, ExtPPI = 0.046, Fig 5A, center, black points). Because AP’s null value is dependent on class size, relative performance of large GO groups can dominate MAP scores. Therefore, we also report the mean per GO group fold change above the null, where smaller GO groups may have a larger influence, yielding mean fold changes above the null of SEA = 4.6, BlastP = 6.9, CoExp = 2.5, and ExtPPI = 2.8 (Fig 5A, right, black points). Together, the raw and fold change performances strongly suggest a high degree of utility in the ligand data. We suspected the drop in performance for the co-expression data compared with its AUROC score is that it is specifically constructed to have no topological outliers through rank standardization and meta-analysis (i.e., every gene is connected by only moderately varying weight to every other gene).

We next looked for edges within the networks that may drive performance. Our concern is that if a single edge, characterizing only two genes, is too informative, it will strongly affect precision-recall but may not generalize to other genes meaningfully. Our approach was to assess the networks for connections of both high weight and high Jaccard overlap in GO annotations (a form of semantic similarity) and therefore likely to contribute to function inference performance. Specifically, we first sparsified the networks to include only the top 1% of interactions for each network (Fig 5A, green points: Sparsified). Here, all networks except ExtPPI fell in AUROC, while all networks except the co-expression network rose in MAP, consistent with the view that AUROCs are dependent on the entire data set and MAPs on high-performing outliers (which co-expression lacks). Then, we additionally sparsified the networks by greedily pruning edges with low Jaccard overlap until the MAP performance reached that of the original dense network (co-expression was excluded because its sparse performance was lower than the original network) (Fig 5A, blue points: Optimized). Strikingly, this yields very small subnetworks with few connections, totaling 27 unique connections in ExtPPI, 227 in BlastP, and 95 in LigNet, mostly pairs and small cliques (Fig 5B, example BlastP network; S3 Table). Further, while MAP performance is retained, these optimized networks show almost no AUROC performance (Fig 5A, blue points).

In summary, while evaluation metrics such as AUROC evaluate the broad functional encoding in the network—properties that are likely to generalize to new contexts—evaluation metrics such as MAP can evaluate immediate utility of some specific functional localization within the networks. We see that under the MAP metric, network performance is largely consistent with AUROC, however this performance is derived from a tiny fraction of the network. In contrast, performance under AUROC is largely independent of outlier edges, so as coverage in the genomic space is increased, our moderate performance trends reported earlier in the paper are likely to generalize.

## Discussion

The principal observation emerging from this work is that functional genomic and ligand similarity are complementary. The networks contain distinct information, in that shared physical interactions among proteins, and protein co-expression, only occasionally predict shared ligand recognition (Fig 1). Correspondingly, many proteins share similar ligand sets but are unrelated by functional genomics. Indeed, even sequence-related targets are, as often as not, unrelated in the ligands they recognize, until such sequence similarity rises to a high level. Combining the two sorts of networks—ligand-based and any one of the functional genomics networks—improves gene function prediction through guilt-by-association.

In the PPI and co-expression networks, only 5 to 8% of protein pairs shared related sets of ligands (Fig 1) (all the proteins do have ligands to relate, this was a criterion of their inclusion in the analysis). The vast majority of protein pairs that were related by functional genomics not only did not share a common ligand, the ligand sets for these targets did not resemble each other. Though there was a higher likelihood that sequence-related targets would share ligands, almost half of sequence-related pairs recognized dissimilar ligand sets. It wasn’t until BlastP E-values of 1e-86 that two proteins were even 80% likely to share ligands, and this was only at a relaxed level of ligand similarity of 1e-5. At a more conservative SEA E-value of 1e-20, one only reaches an 80% confidence that sequence-related targets will share related ligands at BlastP E-values of 1e-180.

Still, it remains true that a very high level of sequence similarity does predict ligand similarity, allowing one to extend ligand chemotypes from one target for which ligands are known to another for which few if any ligands have been characterized. At a ligand similarity E-value cut-off of 1e-20, there are 348 proteins for which few, if any, ligands are known, but that are related to pharmacologically well-studied targets from which ligands may be extended. As limited as the reliable overlap between sequence- and ligand-based similarity is, it could extend our coverage of human proteins for which ligands are known by up to 20%. This study is the first, as far as we know, to quantify the relationship between sequence and ligand similarity, and may provide a useful guide to protein deorphanization, an area of active interest [34–36].

Returning to our main theme, the complementarity (and orthogonality) of the functional genomics and the chemoinformatic networks implies that merging the two adds an unusually high amount of new information to what either can represent alone (Fig 4). Naturally, this is only interesting if we believe that the chemoinformatics associations are themselves meaningful, but this is supported by the correlation of the ligand-linked targets with shared functional terms in GO (Table 1), and by the strong correlation between proteins linked by ligand similarity and by Traceable Author Statements about protein function, which is considered the highest quality annotations in GO.

One route to take advantage of the complementarity between the ligand similarity network and functional genomic networks would be to include ligand similarity as a data source with existing automated gene function prediction methods [37]. The critical assessment of function annotation (CAFA2) recently completed its second evaluation across 126 methods from 55 research groups competing to predict gene function annotations the Gene Ontology and the Human Phenotype Ontology [38]. 53 groups used some form of sequence similarity, 20 used physical protein interaction data, and 12 used gene expression. While the SIAM/FireDB method uses protein-ligand co-crystal structures [39], as far as we are aware, no team used data from ChEMBL or other large protein-ligand database to construct their predictors. This absence is conspicuous since evaluating diverse data sources should have larger impact than a varying inference algorithms [40]. The utility of GBA may be limited if performance is derived from a small subset of edges [33], which can happen if the dataset size is sufficiently sparse. However, this still speaks to evaluating data and not the method itself.

Certain caveats bear mentioning. We have only compared ligand similarity with a few functional genomic and bioinformatic networks; though these are widely used, other networks may correlate more strongly with ligand similarity, or with different ligand similarity metrics. To compare the SEA, co-expression, and extended PPI networks on equal footing, we restricted our analysis to 1,131 proteins, less than half of the number for which ligands are known, and a small fraction of the genes in the genome. In particular, this set contains 353 kinases so that kinase-kinase associations are over represented in the BlastP (86.5%) and LigNet (74.0%) networks and but not in the ExtPPI (10%) and CoExp (14.4%) networks. On the one hand, this means SEA’s results will be biased toward kinases, just as PPI data is biased toward promiscuous proteins [41] but on the other hand it is precisely this sort of variable performance and bias which yields complementarity when the data is integrated [40]. The biases and limitations in the individual data modalities do not detract from the need to improve each data set individually, but speak more to the fact that integrating them is more practical. Expanding within each data modality is a necessary task, for instance through the expansion of target and ligand studies, or by including more co-expression networks within the aggregate network. However, our assessment of the overlapping subset is critical because it demonstrates the complementary functional genomic evidence that can be found across data.

Within each data modality we have chosen straightforward “tinker-free” curation and inference methods to robustly characterize the relationship with ligand-based data. We expect that more sophisticated data curation and inference methods would improve performance over the baseline gene function prediction. On the one hand, this may make all network modalities align more closely because each of them would be better aligned with GO but we suspect the complementary functional value added by including of the SEA network would remain, particularly for novel functional prediction, where fitting to GO (and prior knowledge) does not enhance similarities. In considering improvements on PPI data, for example, filtering out spurious interactions [41], taking into account bias-variance tradeoff in the assay throughput and technique [3, 42], and resolving splice variant specific interactions [43] would be worth exploring. For co-expression data, taking into account conditional variation [44], conservation across species [45], and alternative measure of co-expression [46] would be a possible direction. Correspondingly, for sequence based methods, considering profile-profile sequence similarity [47], domain architecture [48], and ortholog/paralog relationships [49] would be useful to obtain more functional information on the sequences. Finally, to infer gene function from observed functional annotations and these networks, using semantic similarity [50] as a network as well as alternative guilt-by-association machine learning methods [51] could help further understand the novel information within these multiple data modalities. Given that the LigNet network is by itself predictive of gene function, to reduce its contribution would require making the LigNet network predictable from the other networks. So that, while improving the quality of other networks may lead to some concordance within the framework being tested (e.g., GO), the specific complementarities of which functions are predictable by different data modalities (e.g. Table 1) suggests this is not likely to happen. Indeed, we observe that the discordance between distinct data modalities—including the LigNet network—is even clearer when set beside the concordance between different networks built from the same modality (S2 Fig).

These caveats should not obscure the central observation of this study nor its potential applications. Ligand similarity and functional genomic networks complement one another. Functional genomic similarities are largely orthogonal to the recognition of similar ligands among pairs of proteins, and the one cannot be used to predict the other. This independence supports the observation that combining the networks leads to an unusually large increase in accuracy at gene function prediction. Though the exact level of discordance may differ for different measures of ligand and proteomic similarity and evaluation of gene function prediction [52], the value of this under-utilized data resource for functional characterization is likely to remain high.

## Materials and Methods

### Data resources

Genes are identified by the UniProtKB entry truncating the “_HUMAN suffix”. Functional classes are defined by the ChEMBL top-level classification. GO terms (gene_ontology.1_2.obo) were downloaded on 3/12/2013. GO annotations (gene_associations.goa_human) were downloaded on 12/1/2014 and then propagated up the ontological hierarchy. GO terms were filtered for having at least 20 and at most 1,000 annotated genes, over the 1,131 genes in our analysis, yielding 944 terms. The median gene had 52 functional annotations. Ligands and affinity data were downloaded from ChEMBL16, across all species. FASTA sequence data for the proteins was obtained from UniProtKB/Swiss-Prot on 2/26/2014. Protein-protein interaction data was obtained from BioGRID (v3.2.106). Only physical interactions for human proteins were used.

### Ligand set similarity using the Similarity Ensemble Approach (SEA)

The SEA method has been previously described in detail [11]. Briefly, ligands were filtered to have molecular weight less than 550, fewer than 15 oxygen atoms, and fewer than 15 nitrogen atoms. Proteins were compared chemoinformatically by calculating the overall similarity of the ligand sets annotated to them in ChEMBL16. Each ligand was converted into a topological molecular fingerprint, which expresses a ligand as a unique pattern of atoms, bonds, and local topological environments [53]. Here, Extended Connectivity Fingerprints (ECFPs) [54] were used. The similarity between any pair of fingerprint bit strings (ligands) is quantified by the bits they share in common divided by the total number of bits between them, the Tanimoto coefficient (Tc) [55]. The sum of all Tc values, over a cutoff, between all the molecules in the two target-ligand sets is compared to what we would expect for two sets of ligands of the same set size randomly drawn from ChEMBL. The ratio of the observed sum of Tc values to that expected at random is divided by the standard deviation of the random similarity to give a Z-score; when plotted against an extreme value distribution, this gives an expectation value (E-value) [11].

### Network construction

We define a network here as matrix with rows and columns representing the nodes (genes), and the edges between them a value in the matrix (between 0 and 1). A binary network will only contain a value of 1 if there is a relationship between the two nodes. A weighted network, on the other hand, will have a value in between 0 and 1 indicative of the strength of the relationship. For dense networks, every edge is filled with a value, while sparse networks contain 0s. To construct a network, one needs information on all the pairs of nodes specific to each data modality. The BlastP network was generated from sequence similarity E-values computed with default parameters from the FASTA sequences obtained from UniProtKB/Swiss-Prot. An edge was taken as the -log10(E-value). The network was then ranked and standardized to limit the values from 0 to 1.The binary SEA network (LigNet) was generated by filtering SEA similarity associations with E-values < 1e-5 across chordate genes and mapped to human genes via HomoloGene [18]. A weighted SEA network was constructed in a similar fashion to the sequence similarity network, but this time using the –log10(E-values), and then ranking and standardizing the network. The CoExp network was generated by aggregating data from 42 experiments downloaded from Gemma [56], each with a minimum of 10 samples, and at least 17,000 genes detected, totaling 940 samples, and approximately 20 billion reads. Each individual network was constructed by taking the Spearman’s correlation coefficient as an edge weight, which was ranked and standardized. The aggregate network was the sum of all these networks, also ranked and standardized. To model indirect connections in the PPI network [27], we extend the network by adding edges between node pairs with minimum path length less than 7, weighted by the inverse of the minimum path length yielding the ExtPPI network.

### Guilt-by-association

Guilt-by-association (GBA) is a common biological idea used in gene network analysis, which relies on the hypothesis that genes with shared interactions within a network also tend to share common functions i.e., functional annotations from GO or KEGG. GBA is used to assess how correct a network is, with performance measured using cross-validation, in which known functions are masked from part of the network and the ability to recover the information is measured. Given a weighted undirected network *N* over *n* genes represented as a symmetric *n* by *n* matrix, and a set of annotations *A* for *t* terms represented as a (typically sparse) *n* by *t* Boolean matrix, a guilt-by-association predictor propagates annotations in *A* through the network producing a (typically non-sparse) weighted *n* by *t* matrix *P* of predicted annotations. The degree null predictor is:

$$P_{D}\left[i,j\right]=degree\left(N,i\right)$$

The neighbor voting predictor is:

$$P_{NV}=\frac{N*A}{P_{D}}$$

That is, each gene is given a score equal to the fraction of its neighbors (including itself), which possess the functional annotation evaluated. For cross validation, the rows of *A* were split into 3 randomized folds and predicted from the remaining annotations in *A*. Accuracy was assessed for each term as the average AUROC of the withheld annotations by the ranked predicted annotation weight, computed as:

$$AUROC=1-\;\;\frac{\frac{p}{np}-\frac{np+1}{2}}{nn}$$

where *np* and *nn* are the number of true and false withheld annotation and *p*_*i*_ the rank of the *i*^*th*^ gene in that functional term (across all *n* genes). For each predictor, the distribution of per-term scores is plotted as a kernel density estimation using a Gaussian kernel and a bandwidth of 0.05 (Fig 2). Since AUROC is invariant to class distribution it leads to broader interpretability at the expense of statistical power [57].

#### Precision-recall

With all other aspects of the data and evaluation otherwise maintained, precision-recall was calculated in our GBA assessment. For each term *t*, the average precision is given as:

$$AP=\frac{1}{k}\overset{k}{\underset{i=1}{\sum }}\frac{i}{rank_{i}}$$

where *k* is the number of genes, and *rank*_*i*_ the positive rank of the gene in the set as given by the predictor in the GBA analysis. Note that because we treat hidden positives as hidden among all possible unlabeled genes for each fold, the null-precision recall is dependent on the number of folds in cross-validation (three, in our evaluation).

### Network analysis

For the correlation and gene function prediction analyses, only the nodes in the intersection of all four networks were used, totaling 1,131 genes. The discordance between networks was assessed by ranking associations in one network and measuring the cumulative fraction of associations in the other network. Sparse networks were constructed by taking the top 1% of connections in each network, using tied ranks, so exact numbers varied modestly, although this did not alter the qualitative results. Comparative performance and degree of change was similar within a moderate range of tested thresholds, generally rising with increasing sparsity past 0.5% density. To determine optimized networks, we ranked connectivity with each sparse network by semantic similarity and iteratively removed connections until the precision-recall of the networks was matched. Precision-recall of networks with the same range of semantic similarities was calculated by using the same minimum threshold and randomly sampling the same number of interactions as were present in the optimized networks; this would return the optimized networks if the connectivity sampled was required to be from the real networks, and they thus constitute a useful null distribution for the role redundant topology specifically degrades the connectivity within the network. Plots were generated using R [58]. The guilt-by-association method described previously is available in the EGAD R package [59].

## Supporting Information

S1 Fig Coverage of proteomic space by ChEMBL targets and sequence similarity. Using SIMAP 1, Eukaryotic targets annotated in UniProt as of August 2014 were compared with each target in ChEMBL16 having at least 5 ligands annotated with activity ≤ 10 μM (yielding 2,117 targets) using the FASTA/Smith Waterman algorithm. The number of distinct targets retained as a function of the E-value threshold for all targets (panel A) and human targets (panel B) are shown (S1 Table).
(PNG)

S2 Fig Prediction ROC for Verleyen 2015 networks. The (Verleyen 2015)[40] networks are binrary(0/1) networks from different sources. Each of these networks was restricted to the set of 1,131 genes in this study and the main networks (ranked) from this study were used to predict them, reporting enrichment with ROC. The SEA network ROC is overlaid in red. AUROC values are shown in each panel with colors corresponding to the predicted network. ExtPPI and CoexpNet both strongly enrich for the corresponding (Verleyen 2015) PPI and Co-expression networks. BlastP and SEA weakly enrich for (Verleyen 2015) PPI networks and almost not at all for Co-expression networks. The SEA network has little enrichment from BlastP, CoexpNet and ExtPPI networks.
(PDF)

S3 Fig GO term prediction by ontology. Decomposition of GO term guilt-by-association AUROC scores by ontology (extension of Fig 3): (A-C) GO terms by permuted network (Black), degree null (grey), and prediction method (See Fig 3), (D-F) by ontology, (G-I) degree null prediction by ontology.
(PNG)

S1 Table Unliganded human genes with BlastP < 1e-180 to a liganded target in ChEMBL. (XLSX)

S2 Table Top predicted GO terms by network. (XLSX)

S3 Table Summary of GO term GBA performance. (XLSX)

S4 Table Associations in Optimized networks. (XLSX)
