# Supplementary figures and images for: Ligand Similarity Complements Sequence, Physical Interaction, and Co-Expression for Gene Function Prediction
Source: PLoS One. 2016 Jul 28;11(7):e0160098. doi: 10.1371/journal.pone.0160098 (PMC4965129; doi:10.1371/journal.pone.0160098)

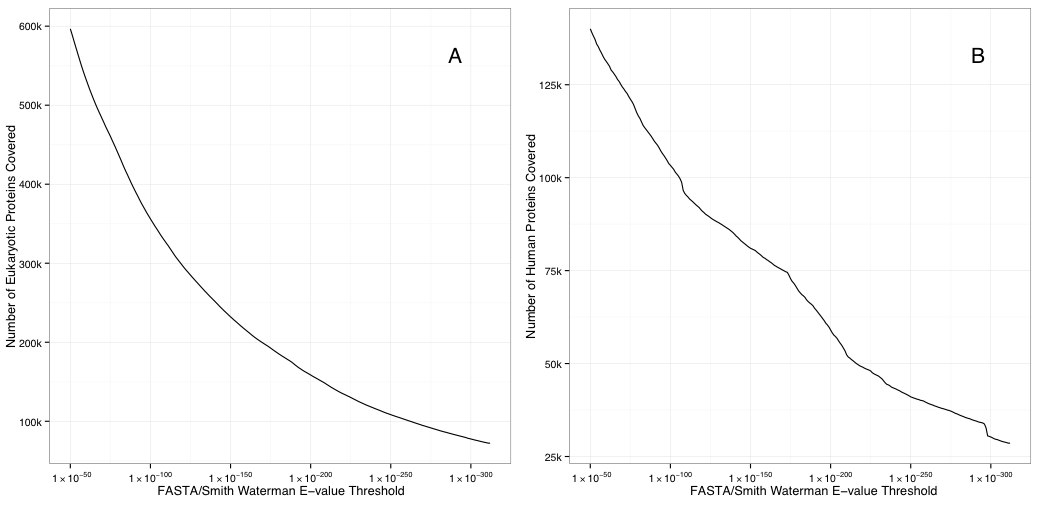

Supplement: S1 Fig — Using SIMAP 1, Eukaryotic targets annotated in UniProt as of August 2014 were compared with each target in ChEMBL16 having at least 5 ligands annotated with activity ≤ 10 μM (yielding 2,117 targets) using the FASTA/Smith Waterman algorithm. The number of distinct targets retained as a function of the E-value threshold for all targets (panel A) and human targets (panel B) are shown (S1 Table). (PNG) [file pone.0160098.s001.png]

# Prediction ROC for Verleyen 2015 networks

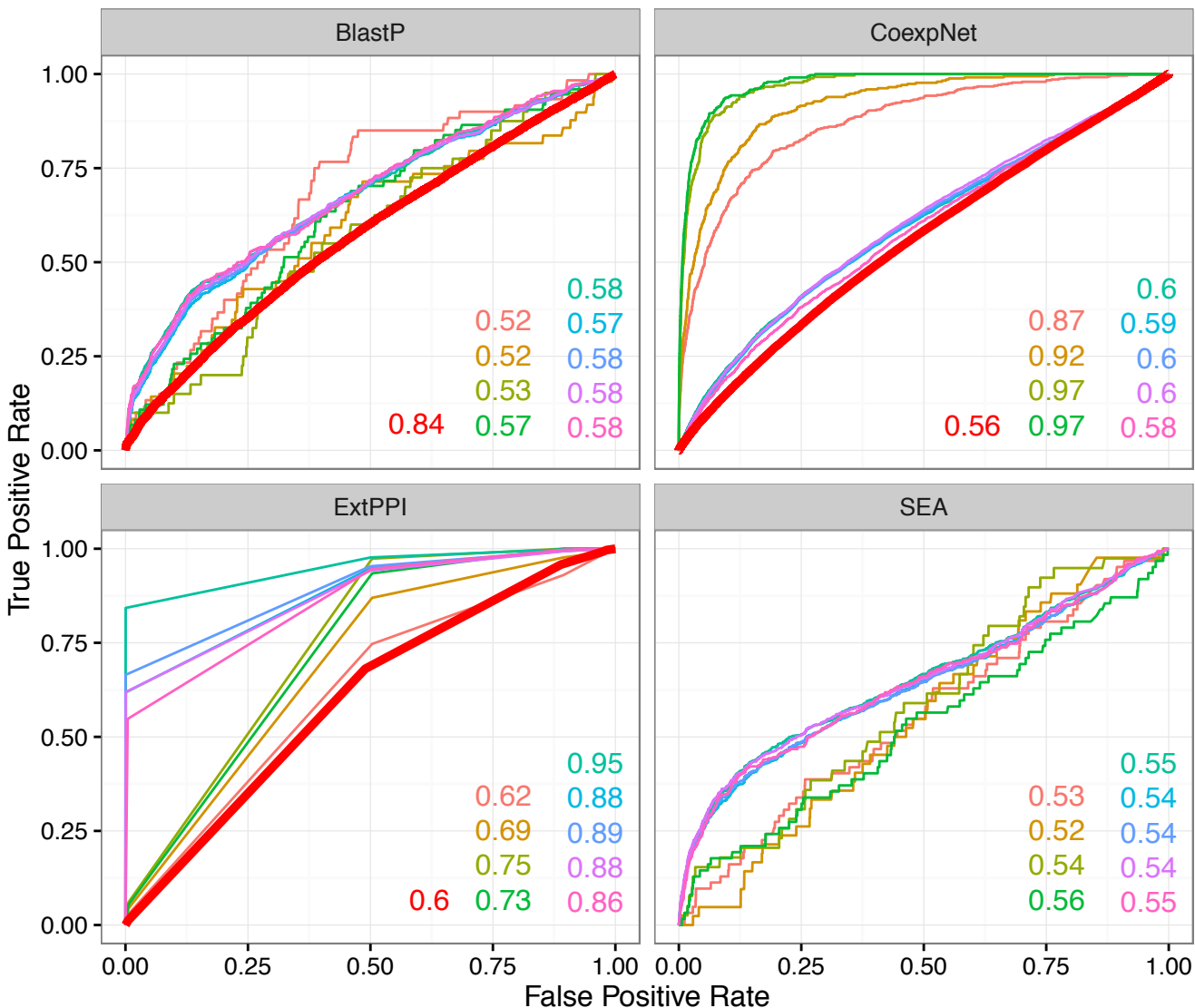

Verleyen 2015  
Networks

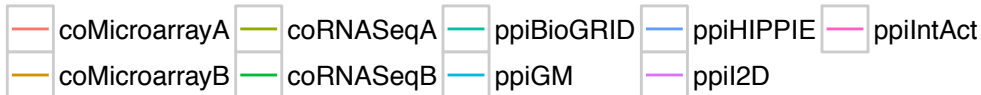

Supplement: S2 Fig — The (Verleyen 2015)[40] networks are binrary(0/1) networks from different sources. Each of these networks was restricted to the set of 1,131 genes in this study and the main networks (ranked) from this study were used to predict them, reporting enrichment with ROC. The SEA network ROC is overlaid in red. AUROC values are shown in each panel with colors corresponding to the predicted network. ExtPPI and CoexpNet both strongly enrich for the corresponding (Verleyen 2015) PPI and Co-expression networks. BlastP and SEA weakly enrich for (Verleyen 2015) PPI networks and almost not at all for Co-expression networks. The SEA network has little enrichment from BlastP, CoexpNet and ExtPPI networks. (PDF) [file pone.0160098.s002.pdf]

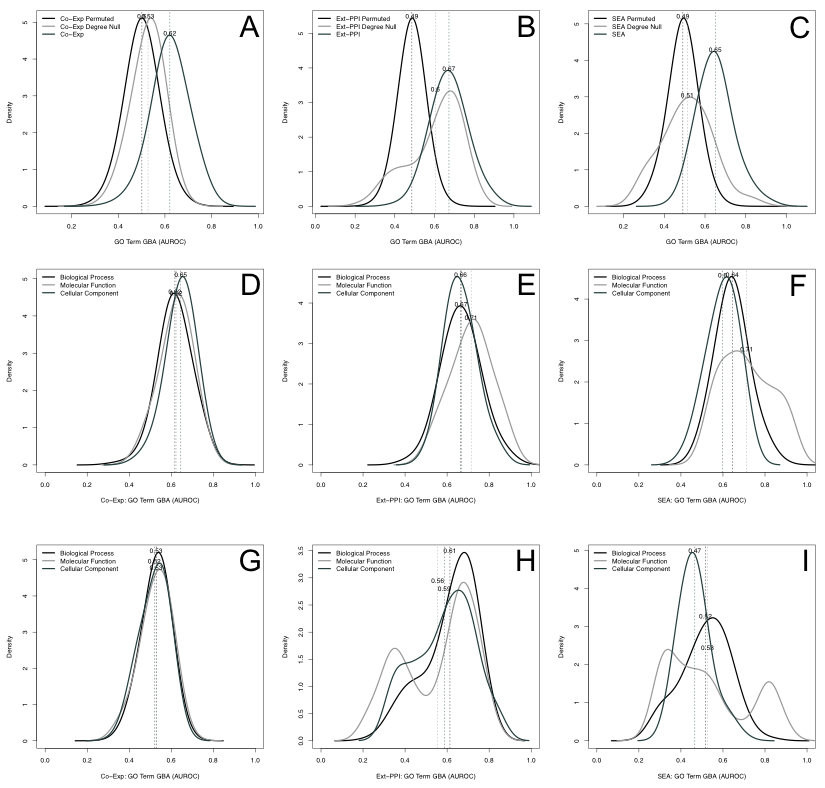

Supplement: S3 Fig — Decomposition of GO term guilt-by-association AUROC scores by ontology (extension of Fig 3): (A-C) GO terms by permuted network (Black), degree null (grey), and prediction method (See Fig 3), (D-F) by ontology, (G-I) degree null prediction by ontology. (PNG) [file pone.0160098.s003.png]
